# Good outcomes of elbow arthroscopy-assisted absorbable screw fixation for capitellum fracture of the humerus in children: a case series

**DOI:** 10.3389/fped.2023.1202537

**Published:** 2023-06-02

**Authors:** Chao You, Zhen Cheng, Yongjie Xia, Chao Deng, Yibiao Zhou, Yiyuan Sun, Guibing Fu

**Affiliations:** Department of Orthopedics, Shenzhen Children’s Hospital, Shenzhen, China

**Keywords:** capitellum fracture of the humerus, elbow arthroscopy, absorbable screw, children, range of motion, clinical results

## Abstract

**Background:**

Capitellum fractures are rare coronal fractures of the distal humerus which accounts for 6% of all distal humeral fractures and only 1% of all elbow fractures. The purpose of this study was to investigate the efficacy and complications of arthroscopically assisted reduction and fixation with absorbable screws for capitellar fracture of the humerus in children.

**Methods:**

This was a retrospective case series study, which considered four patients (4 elbows), ranging from 10 to 15 years old, who were treated by arthroscopic-assisted percutaneous absorbable screw between 2018 and 2020. The elbow flexion-extension and forearm supination-pronation ranges of motion (ROM) were measured at the preoperative examination and last follow-up examination. Finally, the clinical and radiological results were assessed.

**Results:**

The result of operations is satisfactory. The mean follow-up was 3.0 years (range 2–3.8 years). Average range of motion significantly improved from pre- to postoperation, with forearm supination from 60°(50°−60°) to 90°(90°) and pronation improved from 75°(70°−80°) to 90°(90°). The postoperative elbow flexion-extension range of motion was significantly higher compared with range of motion before surgery (*P *< 0.001; *r* = 0.949). At the final follow-up examination, the Mayo Elbow Performance Score was excellent. Satisfactory clinical results were achieved in all patients, and no postoperative complications were observed.

**Conclusions:**

It is an effective and safe surgical option to use arthroscopic-assisted percutaneous absorbable screw fixation for treating capitellum fracture of the humerus without any complications in children.

**Level of evidence:**

Level IV; case series.

## Background

Capitellum fractures are rare coronal fractures of the distal humerus which accounts for 6% of all distal humeral fractures and only 1% of all elbow fractures (1). Capitellar fracture of the humerus is usually classified according to the Morrey classification in adults (2): Type I (Hahn-Steinthal fracture) is common. It refers to a complete fracture of the capitellum, which may be accompanied by a small amount of involvement of the outside of the trochlea of the humerus. This type of fracture can be easily identified on x-ray; Type II (Kocher-Lorenz fracture) is a fracture of thin bone cartilage fragments peeled from the capitellum. Fracture fragments are often not shown on x-ray, which brings certain difficulties to clinical diagnosis. Type III (Broberg-Morrey fracture) is compression or comminuted fracture of the capitellum. Later, after McKee's classification modification, the concept of type IV fracture was given: the coronal fracture of the capitellum extends to the medial side and most of the trochlea of the humerus are involved (3).

The mechanism of injury of capitellar fractures are that the child's palm rests on the ground after a fall, and the external force is transmitted to the elbow along the radius, or when the elbow is fully flexed during the fall, the external force impacts the capitellum through the coronal process of the olecranon, at the same time, valgus stress can cause medial soft tissue damage (4). Due to the inclusion of articular cartilage fragment in the the capitellum fracture of the humerus, and the covering of distal humerus, radiographs are not effective in diagnosis and are easy to be missed.

A large of literature reveals that the main treatment methods for capitellum factures in children include closed reduction (5), excision of the fragment (6), open reduction with internal fixation (5, 7) and arthroscopy assisted percutaneous internal fixation (8). However, due to the low incidence of this fracture in children, only a few articles have studied the arthroscopic reduction in the elbow. Thus, the aim of this study was to evaluate the efficacy and complications of four patients with arthroscopically assisted reduction and fixation with absorbable screws for capitellum fracture of the humerus in children.

## Materials and methods

**Figure 1:**
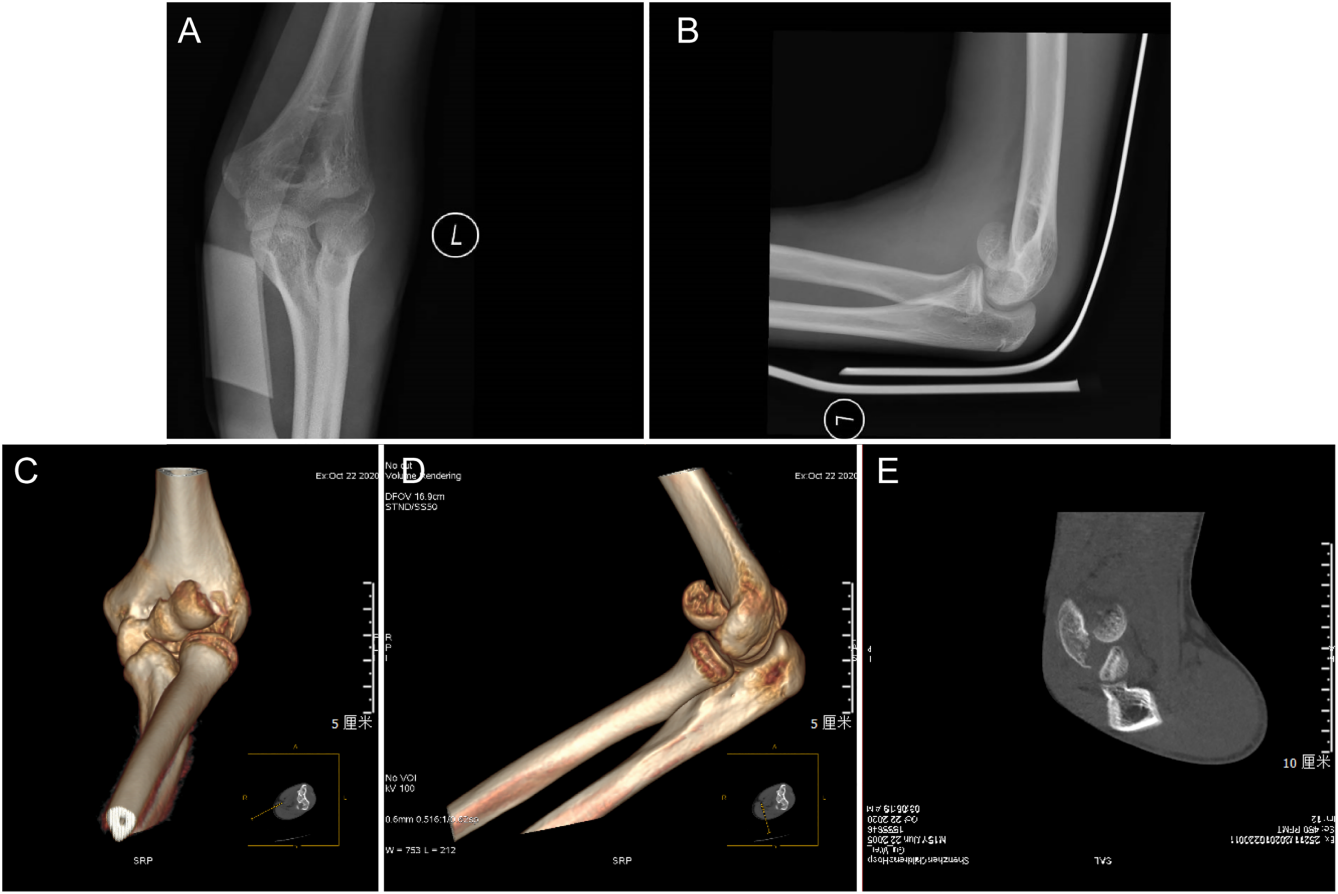
A preoperative radiograph of a 15-year-old boy with capitellum fracture of the humerus.AP (**A**) and lateral (**B**). (**C–E**) The picture shows the CT examination radiographs after injury: CT examination is performed before the operation to show the displacement and relative relationship of the bone fragments.

The study was approved by the Medical Ethics Committee of our hospital, and informed consent was obtained from all patients. In this study, 4 children (3 males and 1 female) with capitellum fracture of the humerus were treated with arthroscopy-assisted percutaneous absorbable screw fixation surgery in our hospital between 2018 and January 2020. The patients’ average age was 12.3 ± 2.2 years (range 10–15.3 years), which included one type I and three type IV capitellar fractures. All patients were evaluated by biplanar elbow radiography and CT scan before surgery (Figure 1). The arthroscopic procedure was performed within 24 h after the injury. All operations were performed by the same senior surgeon.

## Surgical technique

The procedure was performed in the supine position, under the endotracheal intubation anesthesia with the application of a tourniquet and sterile waterproof bandage. The patient's elbow was flexed to 90°, setting the intra-articular pressure as 60mmHg fluid control system. The performing surgeon paid close attention to the extravasation volume of perfusion fluid and soft tissue tension during the operation. Standard arthroscopy portals were created with the arthroscope entering at the position 2 cm proximal to the lateral epicondyle of the humerus and 1 cm anterior to the humerus for better observation of the fracture site.

**Figure 2:**
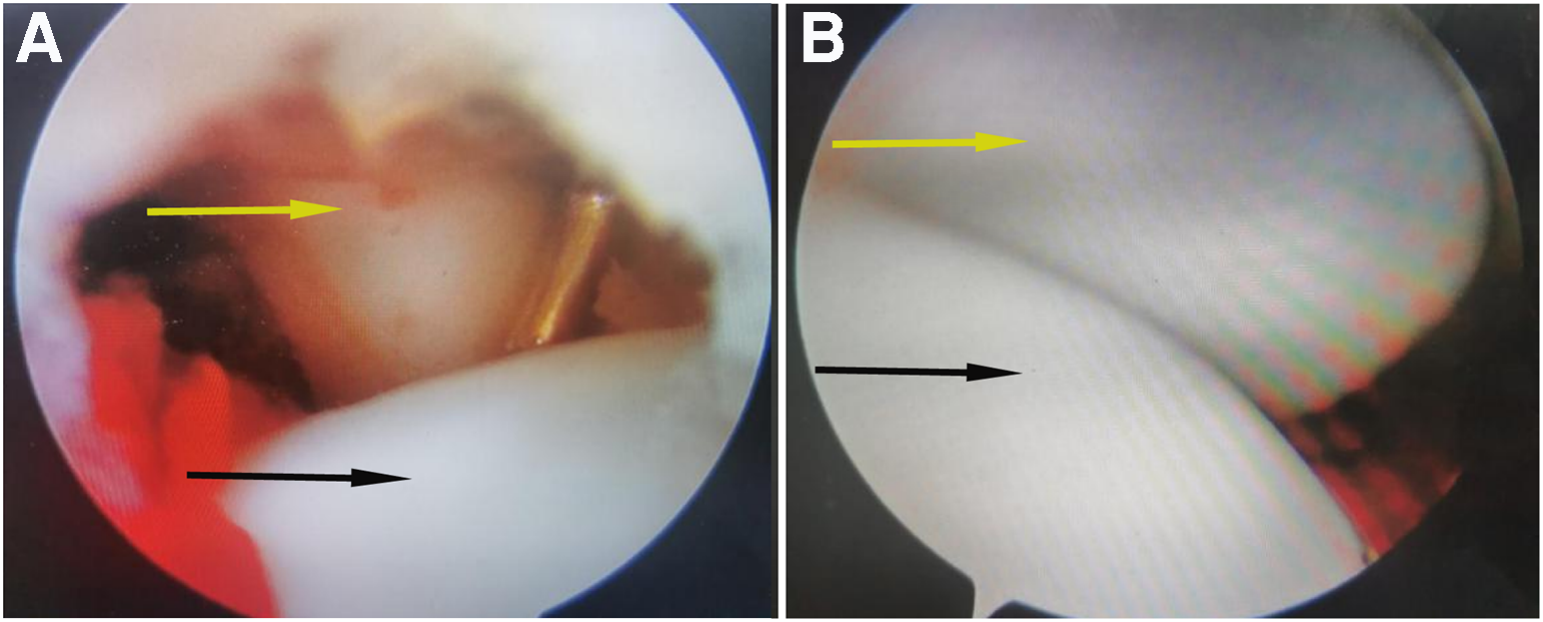
(**A**) Saw the displacement of the anterior part of the humeral head and dislocation of the humeral joint. (**B**) after the fracture was reset. Reduction of the radiocapitellar joint. The picture shows the position and shape of the radiocapitellar joint under arthroscopy before (**A**) and after reduction (**B**). The arrow points to the radial(yellow) and humeral head(black).

First, the hematoma was evacuated with a shaver and the fracture bed was prepared. Intra-articular inspection found the capitellar fragment was displaced anterosuperiorly. To achieve reduction, the fracture fragment is then reduced posteroinferiorly to the anterior surface of the humerus using a hook with the elbow flexed at 30°. The fracture was provisionally fixated using k-wires. Intraoperatively C-arm imaging showed that the guide pin position was satisfactory and the fracture was aligned well. Arthroscopic examination also confirmed anatomic reduction was achieved (Figure 2), and the elbow joint showed satisfactory stability of the osteosynthesis when moved. Later, absorbable cannulated screws were used for fixation with the head buried in the articular cartilage. Diameter, length of screws, and the number of screws implanted were determined considering fracture pattern and the size of the capitellum fragment of the humerus of the elbow. Intraoperative fluoroscopy was used to observe that the tip of the absorbable screw does not penetrate the articular surface. Finally, the incision was sutured and the elbow was immobilized in a long arm cast at a right angle with the forearm in neutral position.

## Postoperative protocol

**Figure 3:**
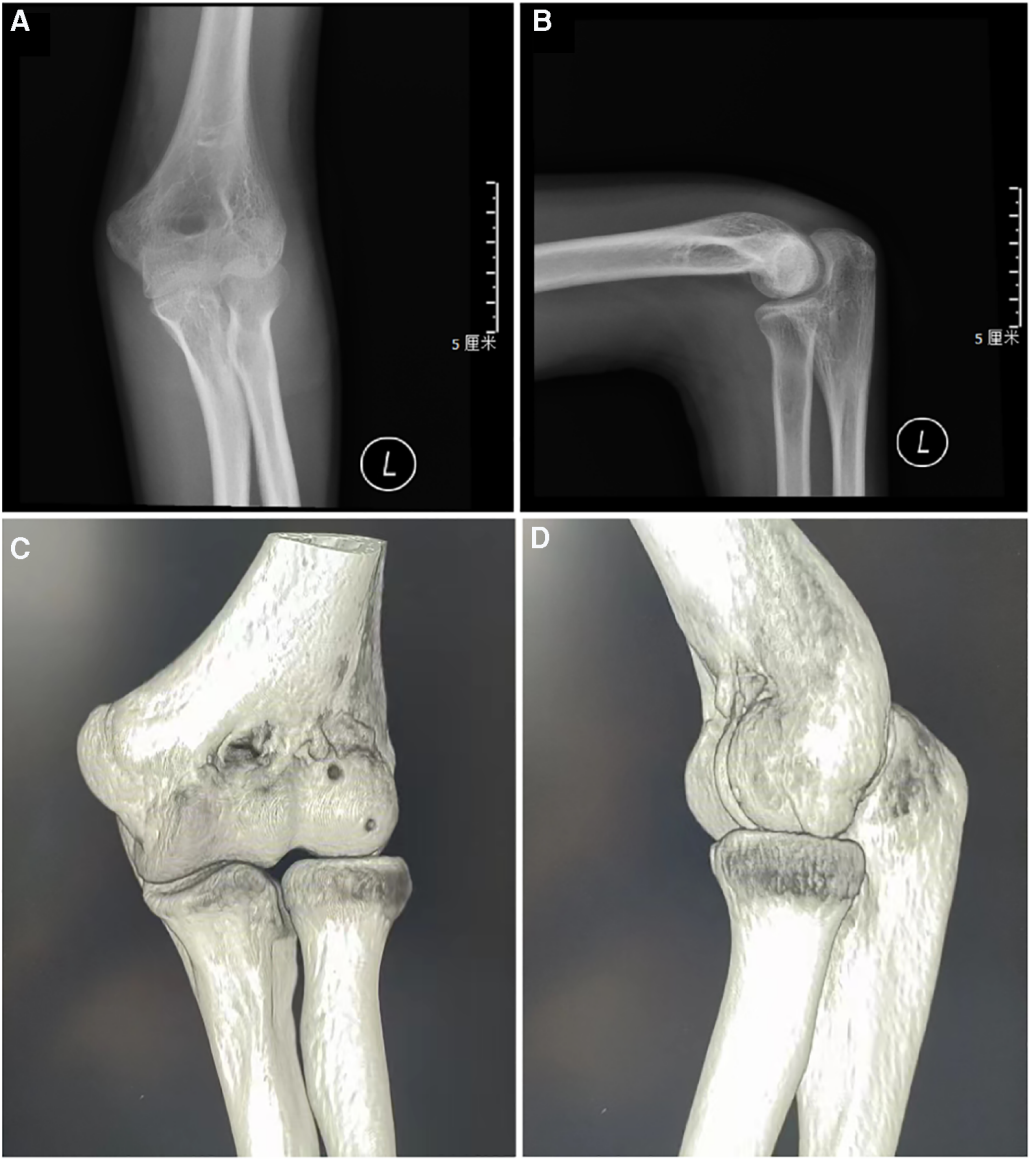
Follow-up: lateral (**A**) and AP radiographs (**B**) after surgery in 4-week later. CT images 2 months after surgery (**C,D**).

**Figure 4:**
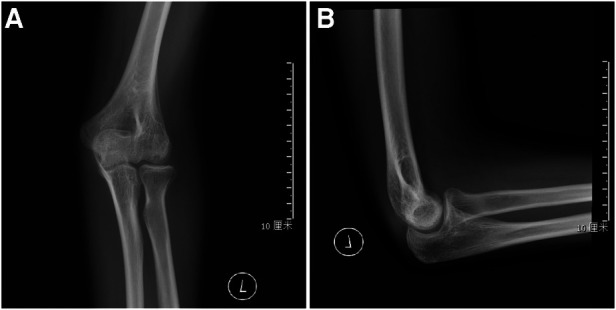
Follow-up: lateral (**A**) and AP radiographs (**B**) after surgery at the final follow-up.

**Figure 5:**
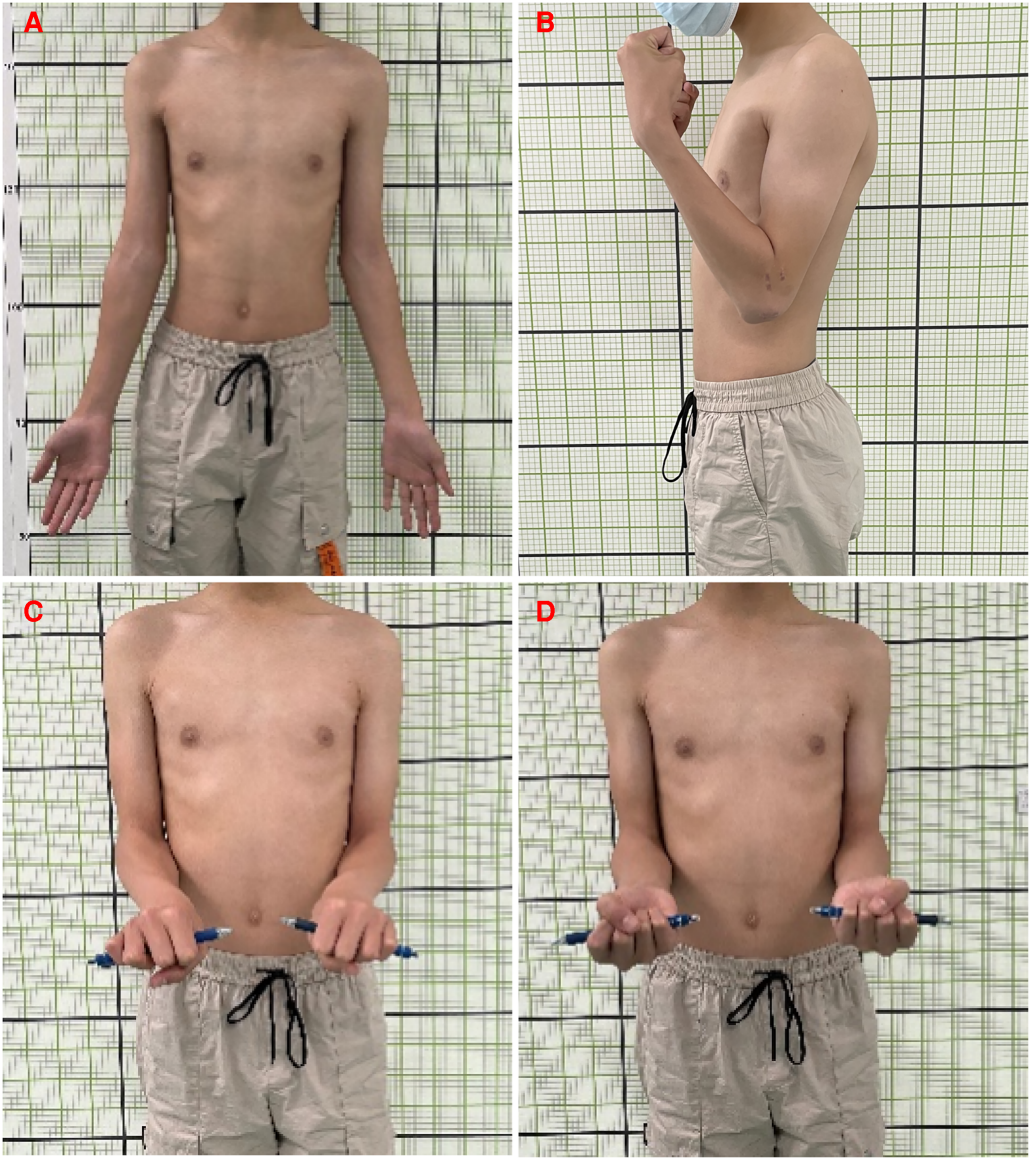
Follow-up: display of elbow joint function after surgery one and a half a year later. (**A**) Extension, (**B**) Extension, (**C**) Supination, (**D**) Pronation.

Postoperatively a long arm cast was applied for one month. Daily routine and functional exercieses were started once the cast was removed. Patients were evaluated at intervals of 1, 3, 6, and 12 months after surgery with clinical and radiological results assessment (Figures 3–5). Thereafter, follow-ups were conducted annually.

## Statistical analysis

We used SPSS (version 26.0, IBM Corporation, Chicago, USA) software for statistical analysis. The data were presented as the mean ± standard deviation or median and inter-quartile range (IQR). Paired t-test was used to compare the data before and after the arthroscopic procedure. *P*-value < 0.05 was considered statistically significant.

## Results

There were 3 boys and 1 girl, with a mean age of 12.3 ± 2.2 years. The patients were followed up at the clinic in the mean duration of 3.0 ± 0.79 years (range 2–3.8 years). The detailed results are presented in Table 1. There were no operative complications, such as nerve injury, compartment syndrome, wound problems, infection, avascular necrosis, elbow deformity or growth disturbances. All the patients were satisfied with the operation results. In all 4 elbows verified successful fixation after reduction of the fracture site by postoperative x-ray or CT. Fracture union was achieved without any additional procedure within 2 months. At the final follow-up, mean of the postoperative elbow flexion-extension ROM (142.5° ± 1.4°) was significantly higher than the preoperative ROM (55.0° ± 4.6°; *P *< 0.001; *r* = 0.949). Although the forearm supination values improved from 60° (50°−60°) to 90° (90°)(*P *= 0.059). Forearm pronation improved from 75° (70°−80°) to 90° (90°)(*P *= 0.063). No statistically significant differences were observed regarding the forearm supination-pronation before and after surgery (Table 2).

**Table 1:** Patients’ demographics and clinical findings of studied patients.

Case No.		1	2	3	4	Mean	SD
Sex		Male	Female	Male	Male	—	—
Age(years)		15.3	10	12	12	12.3	2.2
Operated Side		Left	Right	Right	Left	—	—
Classification		IV	IV	IV	I	—	—
Duration of injury (hours)		8	15	12	10	11.3	3.0
Complications		NO	NO	NO	NO	—	—
Duration of follow-up (years)		3.8	3.5	3	2	3.0	0.79
Elbow ROM (degree)	Pre-op	30–90	40–85	45–95	30–95	36.3–91.3	7.5–4.8
	Post-op	0–145	0–140	0–140	0–145	142.5	2.9
Forearm supination ROM(degree)	Pre-op	0–60	0–50	0–60	0–60	57.5	5
	Post-op	0–90	0–90	0–90	0–90	90	—
Forearm pronation ROM(degree)	Pre-op	0–80	0–70	0–80	0–70	75.0	5.8
	Post-op	0–90	0–90	0–90	0–90	90	—
Mayo Elbow Performance score	Pre-op	<60, poor (or unsatisfactory)	<60, poor (or unsatisfactory)	<60, poor (or unsatisfactory)	<60, poor (or unsatisfactory)	—	—
	Post-op	95∼100, excellent	95∼100, excellent	95∼100, excellent	95∼100, excellent	—	—

**Table 2:** Clinical outcomes at final follow-up.

	Preoperative	Postoperative	*P* value
			pre- vs. postoperative
Elbow ROM(degree)	55.0 ± 4.6	142.5 ± 1.4	<0.001
Forearm supination ROM(degree)	60 (50–60)	90 (90)	0.059
Forearm pronation ROM(degree)	75 (70–80)	90 (90)	0.063

## Discussion

Due to the low incidence and clinical misdiagnosis in the pediatric population, capitellum fracture of the humerus should be treated early because it may limit the child's future elbow function and life. Frank (9) and Taral (6) conducted a retrospective analysis of 3 and 8 adolescents with capitellum fracture of the humerus respectively and emphasized the importance of early diagnosis of fractures on imaging (10). Delay in diagnosis will increase the probability of complications in children (6).

Previous articles have reported multiple surgical options for children. Some studies have advocated the use of screw or Kirschner wire fixation after open reduction of fracture (11–13). However, compared with traditional open reduction, arthroscopically assisted surgery in patients has become increasingly popular. Feldman (14) had reported for the first time two cases of Bryan and Morrey type II capitellum fractures in 1997 and adopted arthroscopic removal of fracture fragments. Good clinical results had been achieved and they believed that for this type of fracture, arthroscopic removal of thin fracture fragments and comminuted fragments seems to be a good alternative to open treatment, which can maintain the advantages of arthroscopy while realizing the fusion of fracture ends. Afterward, Kohji Kuriyama reached the same conclusion as Feldman et al. through the follow-up of 2 cases of capitellum fractures (15). In addition, Hardy (16) and Mitani (17) both proposed the use of arthroscopy-assisted percutaneous screw internal fixation to treat adult capitellum fractures, and in their reported cases, the operation process was successful and the patients recovered well after surgery.

In our study, elbow ROM was regained in all patients at final follow-up, despite cases with limited flexion-extension ROM before surgery. Fractures had no significant effect on pronation and supination function of the elbow, so there was no statistical difference in the results. In our operation, anatomic reduction of fractures can be achieved under direct articular vision, and fixation of the fracture ends with absorbable screws after the guide pin fixes the position is a reliable treatment for children. Articles have been written about this method has less surgical damage, less soft tissue peeling, and does not damage the surrounding blood supply. It can achieve early movement and faster recovery of elbow joint function (18). Furthermore, due to the use of absorbable screws, the second operation is avoided. This conclusion of ours is consistent with the view drawn by Goncalves and others (12). However, there are also reports that the anterior part of the capitellum of a child fracture is thin, and the screw cannot be placed satisfactorily. When the screw itself is placed or compressed, the risk of fragment fracture is greater. But, during our surgery, the probability of this problem seems to be very small.

Recently, Matter-Parrat V et al. (8) studied elbow specimens from 6 fresh cadavers to explore the feasibility of arthroscope-assisted treatment of capitellum fractures. They found that the feasibility was good for type I fractures, and the results were satisfactory during follow-up. However, due to the influence of the thickness of the bone fragments of type II fractures, there are certain difficulties in the use of arthroscopy. Holt et al. also pointed out that the standard for treating distal humeral joint fractures under arthroscopy was that capitellum fractures were not comminuted (19). Arthroscopic treatment does not seem to be suitable for type II fractures. Their research was limited to adults, and whether it had the same effect in children remained to be verified. But in our four clinical cases, arthroscopically assisted treatment of type IV and type I capitellum fractures was feasible with satisfactory clinical outcomes.

The results of four patients show that arthroscopically assisted absorbable screw fixation is a promising treatment and the advantages are obvious. Open surgery is more likely to cause the separated fracture fragments to lose blood supply, causing soft tissue damage, and long-term elbow contracture is prone to occur. However, arthroscopic technology requires higher skills and rich experience for surgeons. Perhaps not all children with capitellum fractures are suitable for surgical and arthroscopic treatment. In future clinical work, we expect to have more cases so that we can explore the fracture types suitable for arthroscopy.

## Conclusions

Our research suggests that arthroscopic-assisted percutaneous absorbable screw fixation is feasible for the treatment of capitellum fracture of the humerus in children. The follow-up shows satisfactory clinical and radiological outcomes. It is worth exploring by clinical pediatric surgeons and may be used as an alternative treatment to open surgery.

## Data Availability

The original contributions presented in the study are included in the article, further inquiries can be directed to the corresponding author/s.
